# Editorial: Horizons in synaptic neuroscience

**DOI:** 10.3389/fnsyn.2023.1295640

**Published:** 2023-10-09

**Authors:** Per Jesper Sjöström

**Affiliations:** Brain Repair and Integrative Neuroscience Program, Department of Medicine, Department of Neurology and Neurosurgery, Centre for Research in Neuroscience, Montreal General Hospital, The Research Institute of the McGill University Health Centre, Montreal, QC, Canada

**Keywords:** astrocyte, neurotransmitter release machinery, hearing loss, co-release, tripartite synapse

## Introduction

I am delighted to present the inaugural “*Horizons in synaptic neuroscience*” Research Topic. This Research Topic showcases high-impact, authoritative, and reader-friendly review articles covering the most topical research at the frontiers of synaptic neuroscience. As Chief Editor, I was asked to identify and nominate the contributing authors in recognition of their prominence and influence in their respective fields. The cutting-edge work presented in this Research Topicthus highlights the diversity of research performed across the entire breadth of the synaptic neuroscience field and reflects on the latest advances in primary as well as translational research.

## Papers in this Research Topic

### Primary research

In the classic view, synapses are communication devices between neurons. However, together with pre- and postsynaptic neurons, astrocytes form structures called tripartite synapses, by which they participate in bidirectional synaptic communication (Perea et al., 2009). Whereas the impact of the transcription factor cyclic adenosine monophosphate (cAMP) response element (CRE)-binding protein (CREB) has been relatively well investigated in neurons, less is known about its role in astrocytes. In their mini review, Kim and Kaang therefore explore how CREB mediates responses in astrocytes. They first discuss the classic G protein-coupled receptor activated cAMP/protein kinase A (PKA) pathway for activating CREB, but they also examine non-canonical pathways, such as receptor tyrosine kinases, Notch, and Phosphatidylinositol 3-kinase/Akt. They end with a brief note on CREB in reactive astrocytes and pathology. In sum, this mini review highlights the significance of CREB in gliotransmission from astrocytes to neurons and their synapses, an important but relatively less well studied topic that deserves considerable further research.

Another classic view is Dale's law, i.e., the notion that a neuron releases the same chemical transmitter from all its synaptic outputs regardless of target cell identity, a postulate that Sir John Eccles was first to attribute to Henry Dale (Eccles et al., 1954). It has, however, long been argued that Dale's principle does not always hold (Sossin et al., 1990; Jonas et al., 1998; Nicoll and Malenka, 1998). The mini review of Kim and Sabatini highlights how neurons that release more than one type of neurotransmitter have been found in many organisms and brain areas. They focus more specifically on how a key challenge with exploring synaptic co-transmission lies in the tools and approaches necessary to understand multi-transmitter release. For instance, it can be difficult to determine whether two transmitters are co-packaged in the same vesicles, or alternatively independently released via distinct vesicles of the same presynaptic terminal. Kim and Sabatini discuss the merits of different methods for addressing such queries, such as proteomics, electrophysiology, optical approaches, and statistics.

### Translational research

Since neurons critically rely on chemical neurotransmission for information transfer, it is not surprising that malfunction of transmitter release is linked to neuropathology. In their review, Uzay and Kavalali discuss how different mutations in various components of the release machinery lead to neurological and psychiatric symptoms, by affecting cross-neuron information transfer and nervous system function. They first explore soluble *N*-ethylmaleimide-sensitive factor attachment protein (SNAP) receptor (SNARE) proteins such as Synaptobrevin-2, SNAP25, and Syntaxin-1, and then move on to investigating the calcium sensor Synaptotagmin-1, the SNARE-stabilizing complexins, the synaptic vesicle fusion protein Munc18-1, and the vesicle priming machinery protein Munc13. The authors conclude that, despite the wealth of knowledge on the synaptic release machinery, there is much to clarify regarding how pathogenic mutations affect release so that we can develop new therapies that are adapted to the distinct dysfunction associated with specific genetic variants.

Astrocytes have also been associated with neuropathology, which is perhaps also expected since they vastly outnumber neurons in the brain (Sofroniew and Vinters, 2010). In their mini review, Wang et al. discuss how astrocytes by way of their key role in neurodevelopment have a major impact on diseases related to intellectual disability. They explore reactive astrocytes, ion channel as well as molecular dysfunction, and the role of astrocytes in environmental factors such as excessive alcohol intake during pregnancy. They end by discussing the effects of intellectual disability drugs on astrocytes, to highlight the potential for therapy. One could thus consider the astrocyte both friend and foe in intellectual disability.

Globally, >1.5 billion people suffer from hearing loss (Chadha et al., 2021). In children, sensorineural hearing loss is the most frequent congenital sensory disorder. Of these cases, 70% have been ascribed to non-syndromic hearing loss (Sindura and Banerjee, 2019). In their review, Li et al. discuss how the GAIP interacting protein c terminus 3 gene *GIPC3* is strongly associated with non-syndromic hearing loss, and how screening for *GIPC3* variants is key to early detection of hearing loss in children. They overview the *GIPC3* gene, explore effects of *GIPC3* mutation on the auditory system, and conclude that focussing on *GIPC3* is useful for understanding hereditary deafness and its developmental mechanisms.

### Concluding remarks

This Research Topic showcases recent high-profile research at the forefront of synaptic neuroscience. I thank the authors for their hard work and kind contributions, and I hope this Research Topic will inform and inspire future research in the field.

## Author contributions

PS: Writing—original draft, Writing—review and editing.
